# Marketing strategies for promoting workplace hepatitis B and C virus testing: a cross-sectional study using correspondence analysis in Japan

**DOI:** 10.3389/fpubh.2025.1522850

**Published:** 2025-03-06

**Authors:** Kosuke Sakai, Shoko Nakazawa, Kota Fukai, Yuko Furuya, Masaaki Korenaga, Masayuki Tatemichi

**Affiliations:** ^1^Department of Preventive Medicine, Tokai University School of Medicine, Isehara, Japan; ^2^Hepatitis Information Center, The Research Center for Hepatitis and Immunology, National Center for Global Health and Medicine, Ichikawa, Japan

**Keywords:** hepatitis virus testing, social networking services, hepatocellular carcinoma, Japan, human resource

## Abstract

**Aim:**

In this study, we aimed to identify the priority group for promoting the implementation of hepatitis virus testing in Japanese workplaces. To this end, we determined (1) the workplace departments interested in hepatitis virus control, (2) the information required to implement testing, and (3) the most effective communication strategies and social networking services (SNS) for disseminating this information.

**Methods:**

We surveyed 2,000 executives and human resources (HR) personnel from various industries in Japan using an online questionnaire. Respondents were enquired about the information required for hepatitis testing, their use of information media and SNS, and their workplace hepatitis testing practices. Data were analyzed using co-occurrence networks, heat maps, and correspondence analysis to visualize the relationships between workplace positions and information needs, media, and SNS.

**Results:**

Education, training, and recruitment personnel, but not top managers, expressed strong interest in hepatitis virus testing and required information on medical facilities, communication, prejudice management, and support plans. These groups frequently use three typical Internet sites as information sources for HR. Business owners were less interested in hepatitis control.

**Conclusion:**

Our findings will contribute to developing social marketing strategies for promoting hepatitis virus control in the workplace. Despite the government’s notice to strengthen measures against viral hepatitis, it may not be receiving enough attention from employers. A possible strategy is to share practical information with education and recruitment staff through HR-focused media. The validation of the effectiveness of this strategy remains warranted.

## 1 Introduction

The World Health Organization has set goals to eliminate hepatitis B virus (HBV) and hepatitis C virus (HCV) by 2030 (1). Japan reportedly has a lower prevalence rate of viral hepatitis than other Asian countries, such as China, Taiwan, and South Korea (2); however, these infections remain the primary causes of hepatocellular carcinoma (HCC) in Japan, with HCV-related HCC accounting for 70% of all cases (3, 4). The number of deaths and mortality rates attributed to HCC have been increasing since 1975; however, since its peak in 2004, a gradual decline has been reported (3). With enhanced measures against hepatitis viruses, the incidence of HBV and HCV infections among newly diagnosed HCC patients has steadily reduced (5). However, reports indicate approximately 480,000 latent HBV carriers and 300,000 latent HCV carriers in Japan (6). Liver cancer, mostly HCC, is the fifth leading cause of death in Japan, after lung, colon, pancreas, and stomach (7).

Hepatitis virus elimination in Japan has been suggested to be achieved by the death of infected individuals, many of whom are older adults? (8). In support of this, a cohort study conducted from 1990 to 2018 in one region of Japan showed that the prevalence of HBV and HCV declined with aging in that region, with few new infections identified before the age of 40 (8). The age of new onset of HCC in Japan has gradually increased from 1996 to 2019 (5). Furthermore, a survey of healthy blood donors revealed that the incidence of HBV infection was 2.78 per 100,000 person-years, while the incidence of HCV infection was 1.86 per 100,000 person-years (9). For more effective and reliable elimination, it is crucial to enhance hepatitis virus screening and ensure timely treatment for individuals who test positive.

In Japan, workplace health checkups have been utilized to identify individuals infected with the hepatitis virus (10, 11). Since the law requires employers to protect the health of their employees, workers in Japan are provided with annual health checkups (12). According to a survey that investigated Japanese workers’ awareness of the hepatitis virus, several untested workers were willing to be tested incidentally during their medical checkup (13). However, not all workplaces conduct hepatitis virus testing during annual health checkups since the hepatitis virus testing is not legally required (14). The Ministry of Health, Labor, and Welfare issued a notice in March 2023 to employers’ organizations and directors of related organizations, requesting their cooperation to eliminate viral hepatitis (15). The government requirements necessitate the development of strategies to promote hepatitis virus control measures among companies.

Social marketing is effective in overcoming global health challenges such as HIV, tuberculosis, and malaria (16). This is a framework for developing public health strategies that adapts the marketing concept of selling products to encouraging consumers to select health behaviors (17). It consists of several steps, the most important of which is segmentation, the identification of the target group to be prioritized based on their demographics and behavioral change preferences (17). Within this framework of hepatitis virus countermeasures, we must perform segmentation to identify the characteristics of the priority population among potential key persons in the workplace for conducting hepatitis virus testing. Adapting social marketing strategies for workplace dissemination of hepatitis virus testing could be an effective approach.

As management and human resource (HR) personnel are responsible for considering health measures in the workplace, encouraging them to proactively work toward controlling viral hepatitis is essential. The social marketing strategy begins by segmenting by understanding their wants, needs, and interests to influence behavior change (18). Studies that investigated workplace measures to control viral hepatitis measures indicate the importance of collaboration with occupational physicians, management of personal information, and addressing stigma about hepatitis (19, 20). However, to the best of our knowledge, no study has identified individuals who require this information and ways to best reach them from a marketing strategy perspective. Correspondence analysis, which visualizes the relationships among categorical variables simultaneously, is effective for segmenting retailers in business marketing; however, examples of its application in social marketing are lacking (21).

The purpose of this study is to segment the potential key persons planning hepatitis virus testing in the workplace and develop tailored strategies for disseminating information regarding implementing hepatitis virus testing in the workplace. Herein, we addressed the following questions: (i) Which departments in the workplace are interested in hepatitis virus control? (ii) What information do these personnel require to implement hepatitis virus testing? (iii) What are the most effective communication media and social networking services (SNS) for delivering this information to these personnel? The results of this study will contribute to increasing the number of companies offering hepatitis virus testing to their workers. This study will also provide an example of how correspondence analysis can be applied to social marketing.

## 2 Materials and methods

### 2.1 Study design and selection of respondents

Using an online questionnaire, we surveyed executives and HR personnel in Japanese companies. The survey was conducted in March 2024 by Cross Marketing Inc. We collected respondents from construction, manufacturing, transport, postal services, and wholesale and retail trade since reports indicate that individuals in these industries are at a higher risk of contracting viral hepatitis (22). The target population was 400 business owners, 400 HR and administrative staff in high-risk industries, 600 business owners, and 600 staff in other industries. Among HR and administrative staff, our target population consisted of those in a position to review medical diagnostic documents submitted to the company by employees or who managed labor relations. The survey was conducted until 2,000 valid responses were received following the above inclusion criteria. The survey was conducted anonymously to protect the privacy of respondents and to collect honest responses.

### 2.2 Assessment of information required for hepatitis virus testing

We investigated the information required to implement hepatitis virus testing at the workplace. The question was “If you were planning to conduct hepatitis virus testing at your company, what information would help you plan the test?” The options were following items: (a) List of medical facilities and health screening centers where hepatitis virus testing is available; Testing agency, (b) Frequency and timing of hepatitis virus testing; Testing information, (c) List of medical institutions where you can be examined when you are found to be infected with hepatitis virus; Medical facilities, (d) How to treat an employee who is found to be positive for the hepatitis virus test; Communication, (e) The impact on work when treating for viral hepatitis; Job impact, (f) How to store the results of medical examinations, including hepatitis tests; Result storage, (g) How to protect personal information such as hepatitis test results; Privacy, (h) How to deal with prejudice against the hepatitis virus; Prejudice Management, (i) How to create a support plan for balancing hepatitis treatment and work; Support plan, (j) How to create an in-house system for balancing hepatitis treatment and work; Treatment and Work Balance System, (k) Nothing in particular, and (l) Unknown. To obtain consistent responses, our questionnaire system was designed such that if the respondents selected (k) Nothing in particular or (l) Unknown, they could select no other options.

### 2.3 Assessment of information media and social networking services

Respondents were enquired about the information media and networking services that they regularly used. The question was, “This question is not limited to hepatitis information, but which of the following sources of information do you use to obtain information for your business?” The respondents were asked about certain types of information media, such as NIKKEI, Rodo Seisaku Jihou, Jinji-bu of Japan, HR Pro, Recruit Works, HR Mikata, BizHint, and YouTube, and SNS, such as LINE, Facebook, Instagram, and X (Twitter). For each item, respondents were asked to select one of four options: regular, irregular, only when necessary, or not at all.

### 2.4 Other assessments

The respondents were asked to indicate their sex, age, position, industry, company size, and hepatitis virus testing practices in the workplace. The position was reported from the following options: business owners, labor relations, recruitment education and training, general affairs, and others. To categorize industries, we used the Japan Standard Industrial Classification, which includes 19 options: agriculture and forestry; mining and quarrying of stone and gravel; construction; manufacturing; electricity, gas, heat supply, and water; information and communications; transport and postal services; wholesale and retail trade; finance and insurance; real estate and goods rental and leasing; scientific research, professional and technical services; accommodations, eating, and drinking services; living-related and personal services and amusement services; education, learning support; medical, healthcare, and welfare; compound services; services not elsewhere classified; government, except elsewhere classified; industries unable to classify. The company’s size was categorized as <10, 10–99, 100–499, 500–999, ≥ 1,000, and unknown. Current hepatitis virus testing practices in the workplace were categorized as follows: all employees, employees over 40 years old, preferred employees, new employees, others, not implemented in the company, and unknown.

### 2.5 Statistical analysis

The analysis was conducted in three steps. First, we developed a co-occurrence network that determined the relationship among the required information. We identified the combination of information required for implementing hepatitis virus testing in the workplace. The Jaccard coefficient was used as a measure of co-occurrence (23). The Jaccard coefficient is an index that evaluates the strength of the association between categorical variables. The Jaccard coefficients for Categories A and B were calculated using the number of individuals who selected Category A or B in the denominator and the number of individuals who selected both in the numerator. When the association between categories was strong, the indicator was close to 1; when weak, it was close to 0. Second, we visualized the relationship between the positions and the information. We identified the information required by the target population for each position regarding hepatitis virus testing. Heat maps and corresponding analyses were used for visualization. In the heat map, the degree of bias in selecting information regarding hepatitis virus testing was visualized by calculating the Pearson residuals. In the correspondence analysis, the bias in selecting hepatitis virus testing information for each position is shown in the Figure. Correspondence analysis was classified as multivariate analysis and selected when the variables were categorical data. This method aids in visually understanding the relationships among categorical data, which can be represented by a two-dimensional scatter plot (24). Correspondence analysis is actively used in marketing research to analyze the relationship between product purchase data and customer characteristics; however, it is not frequently used in public health research (21, 25).

In this step, we visualized the relationship between the company’s position and the required information regarding the hepatitis virus. The scatter plots show that the information that stretches from the origin toward the position is more biased and is selected for that position.

Third, we performed a correspondence analysis that revealed the information media and SNS used specifically for each position. Identifying where information is usually obtained will contribute to selecting the media to disseminate information. To create a categorical variable for each information medium and SNS, we assigned a value of 1 when respondents indicated regular or irregular use and 0 when used only when necessary or not at all. We used correspondence analysis to visualize the relationship between positions, information media, and SNS routinely used. We determined that the information media and SNS that stretch from the origin in the direction of the position are more biased and were selected for that position. Statistical analysis was performed using the KH-coder (ver. 3.02c official package, SCREEN advanced system solutions Co., Ltd).

### 2.6 Ethics statement

Informed consent was obtained from all respondents prior to their participation in the survey. The study’s objectives, protocols for managing personal information, and the absence of penalties for nonparticipation were clearly explained. This study was approved by the Ethics Committee of the Tokai University (No. 24105).

## 3 Results

Table 1 lists the respondents’ characteristics. Participants included 1,607 (80.4%) men and 386 (19.3%) women. The largest age group was 50–59 years with 667 (33.4%) respondents, and the smallest was 20–29 with 67 (3.4%). Regarding positions, 1,000 (50.0%) were business owners, 359 (18.0%) were in labor relations, and 373 (18.7%) were in recruitment. In terms of industry, 341 (17.1%) accounted for manufacturing, 256 (12.8%) for services not elsewhere classified, 196 (9.8%) for wholesale and retail trade, and 186 (9.3%) for information and communication. Regarding the testing status, 902 (45.1%) were not implemented in the company, and 487 (24.4%) were implemented targeting all employees. Regarding necessary information, 687 (34.4%) respondents selected testing agencies, 600 (30.0%) selected testing information, and 431 (21.6%) selected unknown. The distribution of responses to necessary information, information media, and networking services by position is shown in Supplementary Table S1.

**Table 1:** Characteristics of participants (*N* = 2,000).

	*N*	%
Sex		
Males	1,607	80.4
Females	386	19.3
Other	7	0.4
Age, years		
20–29	67	3.4
30–39	217	10.9
40–49	406	20.3
50–59	667	33.4
60–69	643	32.2
Position		
Business owners	1,000	50.0
Labor relations	359	18.0
Recruitment	373	18.7
Education and training	98	4.9
General affairs	137	6.9
Other	33	1.7
Industry		
Agriculture, forestry, and fishing	14	0.7
Mining and quarrying of stone and gravel	9	0.5
Construction	171	8.6
Manufacturing	341	17.1
Electricity, gas, heat supply and water	47	2.4
Information and communications	186	9.3
Transport and postal services	92	4.6
Wholesale and retail trade	196	9.8
Finance and insurance	123	6.2
Real estate and goods rental and leasing	159	8.0
Scientific research, professional and technical services	86	4.3
Accommodations, eating and drinking services	48	2.4
Living-related and personal services and amusement services	51	2.6
Education, learning support	56	2.8
Medical, health care and welfare	143	7.2
Compound services	22	1.1
Services not elsewhere classified	256	12.8
Government, except elsewhere classified	0	0.0
Industries unable to classify	0	0.0
Size of company (number of employees)		
< 10	498	24.9
10–99	467	23.4
100–499	442	22.1
500–999	165	8.3
≥ 1,000	418	20.9
Unknown	10	0.5
Employees subject to testing		
All employees	487	24.4
Employees over 40 years old	160	8.0
Preferred employees	175	8.8
New employees	37	1.9
Other	7	0.4
Not implemented in the company	902	45.1
Unknown	232	11.6
Necessary information		
Testing agency	687	34.4
Testing information	600	30.0
Medical facilities	464	23.2
Communication	477	23.9
Job impact	423	21.2
Result storage	277	13.9
Privacy	263	13.2
Prejudice management	280	14.0
Support plan	249	12.5
Treatment and work balance system	240	12.0
Other	5	0.3
Nothing in particular	375	18.8
Unknown	431	21.6
Information media and social networking services		
NIKKEI	813	40.7
Rodo Seisaku Jihou	364	18.2
Jinji-bu of Japan	371	18.6
HR Pro	260	13.0
Recruit Works	250	12.5
HR Mikata	304	15.2
BizHint	216	10.8
Youtube	553	27.7
LINE	968	48.4
Facebook	589	29.5
Instagram	616	30.8
X (Twitter)	592	29.6

**Figure 1:**
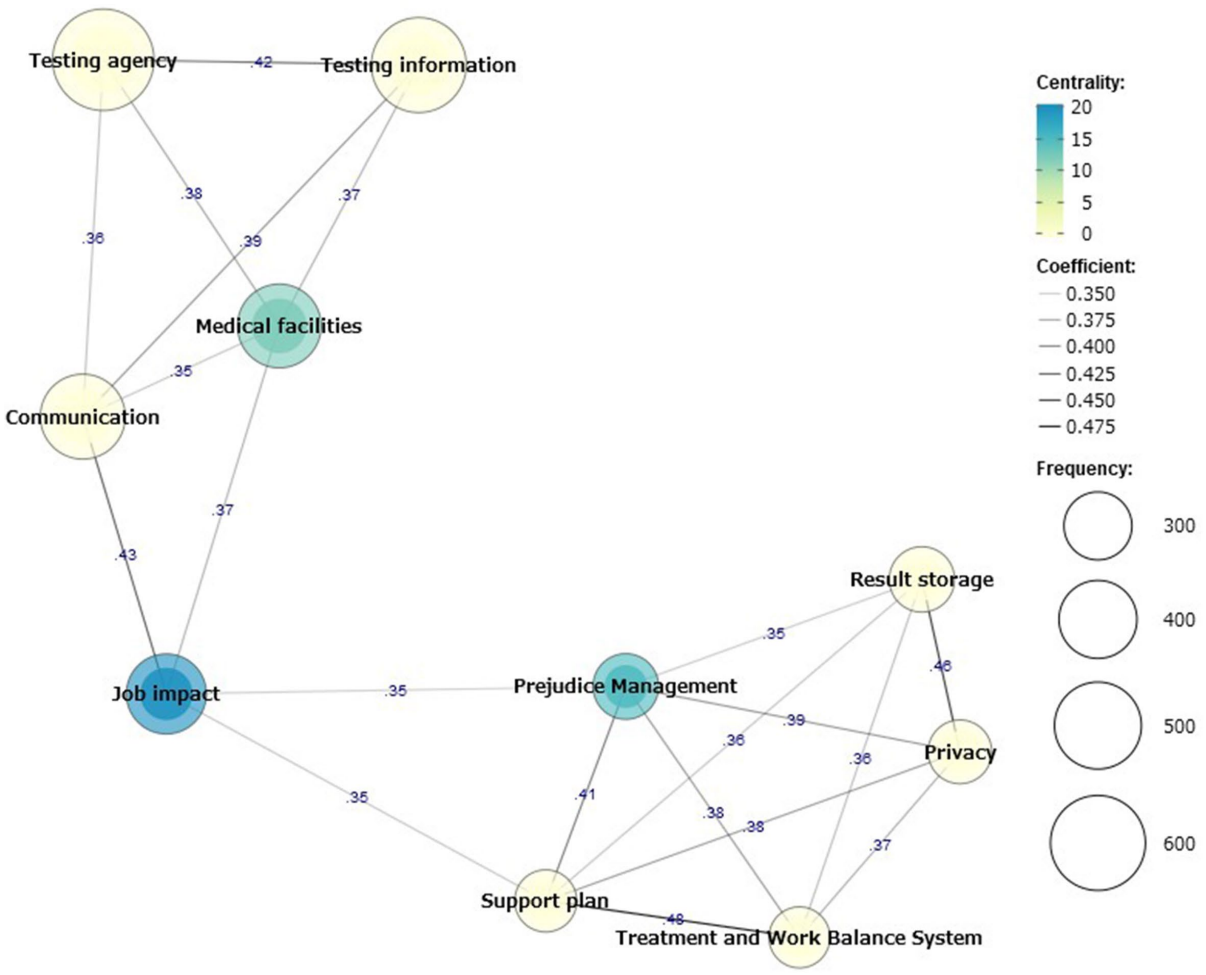
Co-occurrence network of necessary information for hepatic virus testing. Co-occurrence network indicates the strengths of the combinations selected for the required information. The size of the circle indicates the selection frequency, while the color is a measure of centrality, indicating the mediating strength of the overall association of the information. The Jaccard coefficient is the numerical value of the line connecting each piece of information. The Jaccard coefficients for Categories A and B were calculated using the number of people who selected Category A or B as the denominator and those who selected both Categories A and B as the numerator. We found two clusters: testing agencies, testing information, medical facilities, and communication; result storage, privacy, prejudice management, support plan, treatment, and work balance system.

Figure 1 illustrates the co-occurrence network for the information necessary for a hepatitis test. The Jaccard coefficients for all information are listed in Supplementary Table S2. Two clusters were identified in the co-occurrence network: a cluster of testing agencies, testing information, medical facilities, and communication; a cluster of result storage, privacy, prejudice management, support plan, treatment, and work balance system. In the co-occurrence network, information that constitutes many connections in the overall choice is shown in blue, indicating high centrality. Medical facilities in the former cluster showed high centrality, whereas prejudice management showed high centrality in the latter cluster. The size of each circle indicates the selected frequency.

**Figure 2:**
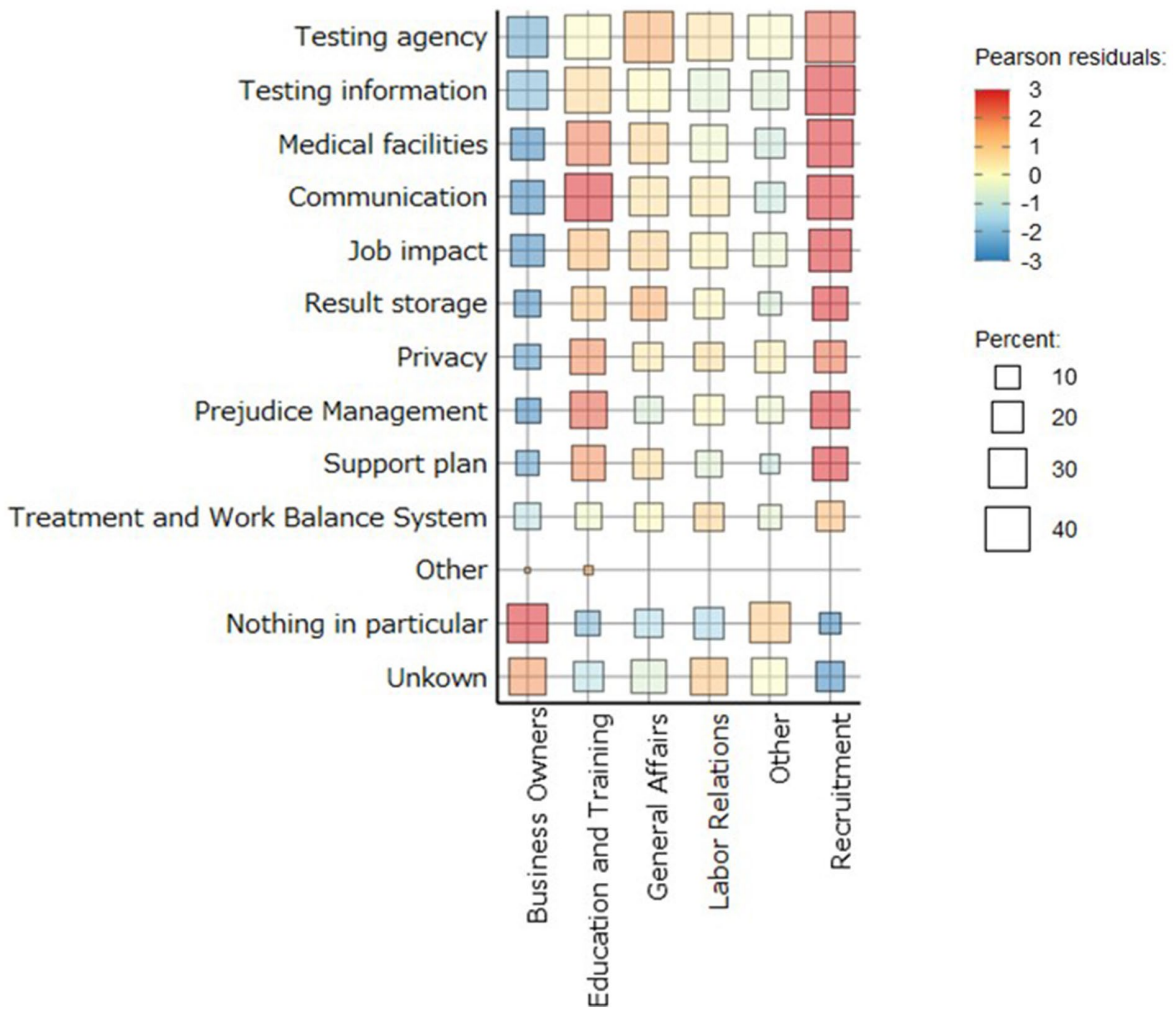
Heatmap of positions and necessary information for hepatic virus testing. Heat map shows the degree of bias for each position in selecting each information in Poisson residuals. For business owners, nothing in particular and unknown are indicated in red. In recruitment, education, and training, medical facilities, communication, job impact, result storage, privacy, prejudice management, and support plan were red. This indicates that the choices were biased in each position.

Figure 2 shows the relationship between the position and information necessary for implementing hepatitis virus testing. The horizontal axis visualizes the bias in choices regarding the necessary information, ranging from blue to red, while the vertical axis represents the percentage of people who selected each option for the necessary information based on their position. The color indicates the Pearson residuals. The size of the squares indicates the percentage of each information choice in the position. Nothing in particular, and unknowns were red to business owners, which indicates that the choices were biased in business owners. Medical facilities, communication, job impact, result storage, privacy, prejudice management, and support plans are highlighted for recruitment, education, and training. In other words, respondents in recruitment, education, and training selected the necessary information regarding the hepatitis virus.

**Figure 3:**
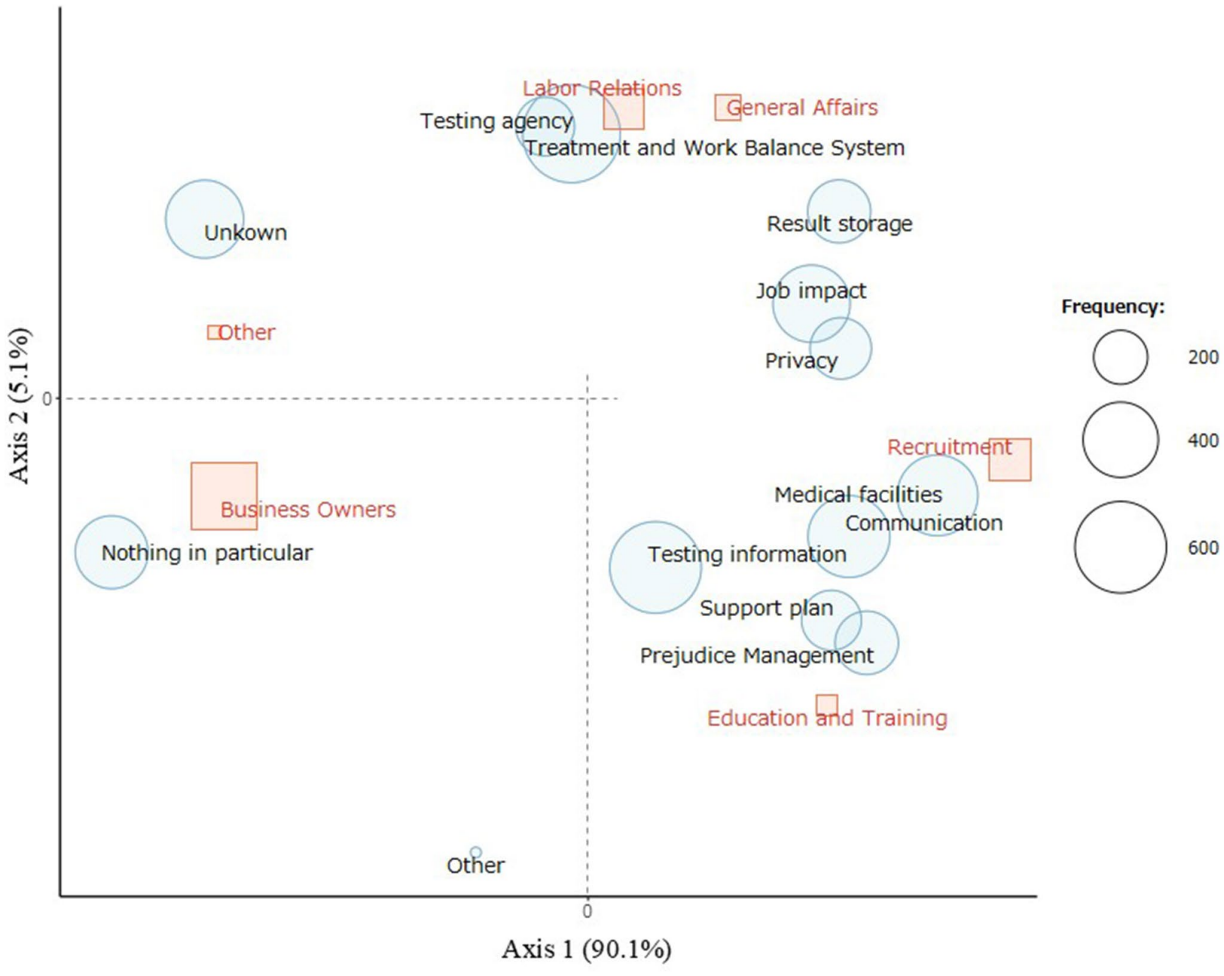
Correspondence analysis of positions and necessary information for hepatic virus testing. Correspondence analysis indicates the information required for each position. Information in the direction of the position from the origin means that it was specifically selected for that position. Nothing in particular and unknown were positioned in the direction of business owners from the origin, which indicates that many business owners predominantly selected them. Medical facilities, communication, prejudice management, and support plans were aligned with recruitment, education, and training, indicating that respondents in recruitment, education, and training specifically selected these options as necessary information. The size of the red squares represents the number of participants in each position.

Figure 3 shows the correspondence analysis results of the positions and the necessary information for hepatic virus testing; the information options specifically selected for each position. Nothing in particular or unknown, was located in the direction of business owners from the origin. This implies that many business owners select nothing in particular or unknown. Medical facilities, communication, prejudice management, and support plans were positioned toward recruitment, education, and training.

Respondents in recruitment, education, and training selected medical facilities, communication, prejudice management, and support plans.

**Figure 4:**
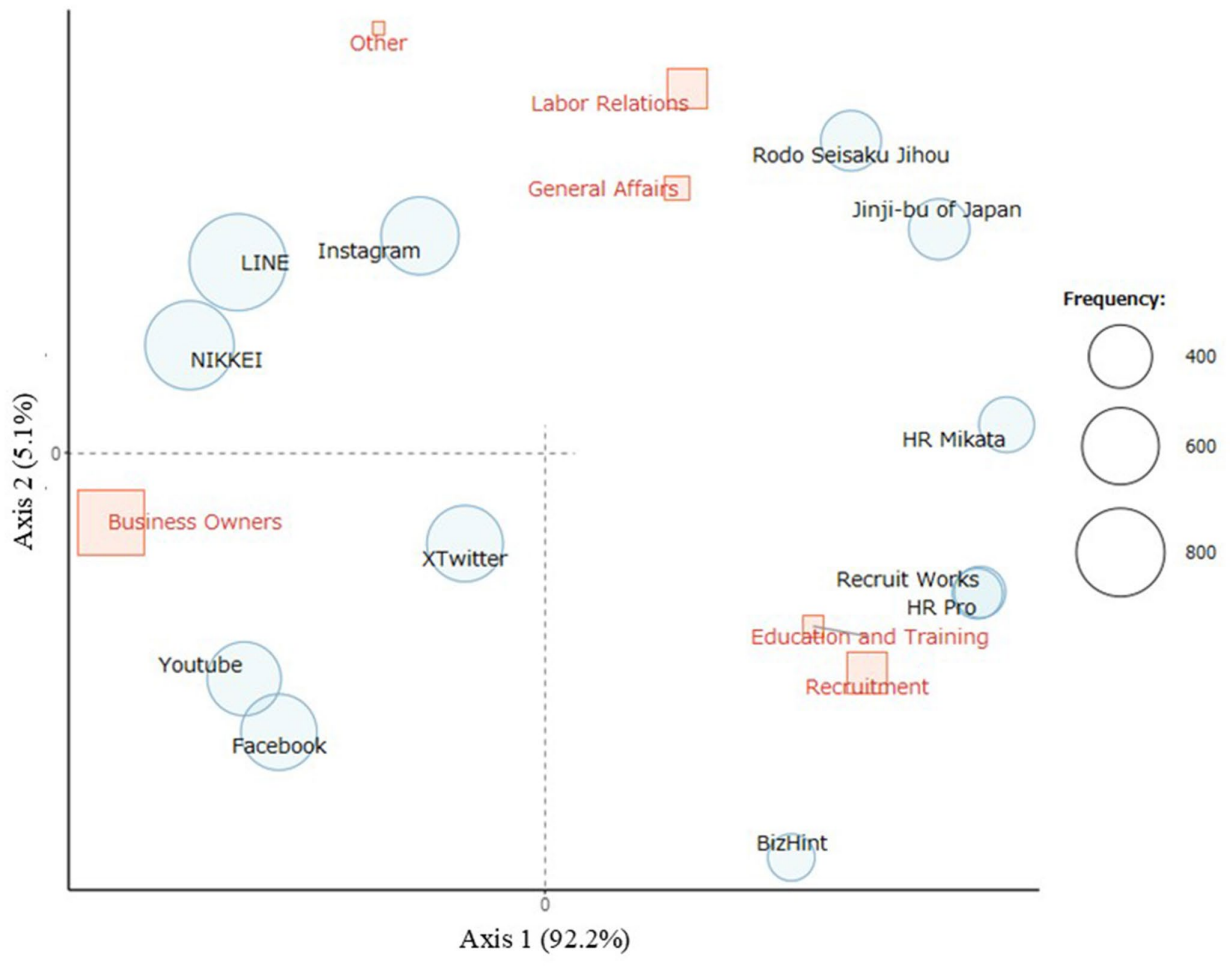
Correspondence analysis of positions and familiar media/SNS. Correspondence analysis shows the familiar information media and social networking services (SNS) associated with each position. Information media and SNS positioned further from the origin in the direction of a particular position can be interpreted as being specific to that position. Recruit Works, HR Pro, and BizHint were preferred by those involved in recruitment, education, and training. The size of the red squares represents the number of participants in each position.

Figure 4 shows the results of the correspondence analysis between positions, information media, and SNS. The HR Pro, Recruit Works, and BizHint options were positioned toward recruitment, education, and training. People in these positions read HR Pro, Recruit Works, and BizHint more often.

## 4 Discussion

In this study, we identified key individuals capable of initiating hepatitis virus testing in the workplace and developed tailored strategies for disseminating information regarding its implementation. The answers to the research questions are as follows: (i) Which departments in the workplace are interested in hepatitis virus control? Departments related to education, training, and recruitment showed interest, whereas management did not. (ii) What information do these personnel require to implement hepatitis virus testing? The key information included is (a) medical facilities that provide hepatitis virus testing, (b) communication strategies for employees who test positive, (c) approaches to managing prejudice against hepatitis, and (d) methods for supporting employees in balancing treatment and work. (iii) What are the most effective communication media and SNS for delivering this information to these personnel? HR websites, such as Recruit Works, HR Pro, and BizHint, which these professionals regularly use. We identified populations that should be prioritized for reaching out through this segmentation. This knowledge could be used to develop strategies to strengthen hepatitis virus control in the workplace with social marketing. We were also able to present a case study of the application of correspondence analysis to segmentation in public health. This method will be useful for other studies to visualize the relationships among many categorical variables.

To enhance hepatitis virus control measures, targeting those in the education and recruitment departments may be effective. Although the government has issued a notice to strengthen viral hepatitis control, it may not have caught the attention of employers. Studies on promoting cancer screening indicate that employers’ interest is associated with implementing workplace screening for gastric, colorectal, and lung cancers (26). Researchers have identified employers and health managers as key for workplace smoking cessation (27). However, in this study for hepatitis virus control, management showed relatively less interest, whereas personnel in the education and recruitment departments showed more engagement. As hepatitis virus testing involves concerns about discrimination and prejudice, recruitment and education staff may be more attuned to this issue than employers. Moreover, although cancer screening and smoking cessation are relatively straightforward health objectives, viral hepatitis may affect only a proportion of workers and remain asymptomatic for extended periods. Consequently, in the business context, managers may perceive viral hepatitis control as a less urgent issue.

Individuals interested in hepatitis virus control require information regarding medical facilities. Studies have indicated the effectiveness of improving access to health care to combat the hepatitis virus (28, 29). According to a study that investigated barriers to HBV and HCV testing among UK immigrants, the disparities in HBV and HCV screenings can be attributed to lack of awareness, language barriers, and challenges in accessing healthcare (30). Information on identifying medical facilities that can treat hepatitis is essential for guiding individuals to post-test treatment.

Communication and prejudice management are important for hepatitis virus control in the workplace, consistent with previous findings (19). According to a study among Japanese occupational physicians and workers, more than half of the occupational physicians experienced responding to consultations regarding hepatitis from employees, and some workers were concerned about work-induced hepatitis exacerbations (19). In another survey on workers’ attitudes, 36% expressed concern about the risk of infection if a co-worker tested positive, 32% indicated they would prefer to avoid contact with individuals with viral hepatitis whenever possible, and 24% admitted they would hold prejudices against a co-worker with viral hepatitis (13). Therefore, employees should be provided with correct information regarding viral hepatitis before introducing the testing in the workplace. To ensure that employees with viral hepatitis are not prejudiced, explaining the results of hepatitis virus tests directly to the workers, without involving managers, proved effective (31).

The findings of this study helped clarify appropriate information dissemination methods. Global efforts to spread awareness about the hepatitis virus have been made, and their effectiveness has been evaluated. In China, a four-week intervention that actively utilized digital crowdsourcing to distribute videos and infographic pictures on the WeChat platform was conducted to educate people about the hepatitis virus, transmission risks, and the need for testing. This approach was effective in promoting testing for viral hepatitis and stigma (28). Conversely, a randomized controlled trial indicated that a crowdsourced intervention regarding HBV and HCV for men who have sex with men did not result in any behavioral change regarding HBV or HCV testing (32).

The dissemination of information that leads to early diagnosis and treatment behavior change is an important issue, and so is the question of the target population in the fight against the hepatitis virus. Currently, the target population is often considered to be a high-risk group. The Test4HepC campaign conducted in the United States, a program that uses social media and a website to promote HCV testing, targeted the generation born between 1945 and 1965, who account for three-quarters of all HCV infections in the country (33).

The multilingual ‘Know Hepatitis B’ campaign conducted in the United States targeted Asian Americans and Pacific Islanders, who account for more than half of all infections (29). According to a systematic review of methods to increase the provision and uptake of testing in Europe, several initiatives targeted high-risk groups such as immigrants, drug users, prisoners, and pregnant women, using settings such as communities, primary care, and hospitals (34). In this study, a survey was conducted focusing on industries in Japan considered high-risk groups, and other target groups were identified based on factors such as the respondents’ interests, needs, and affinities. This study is an important milestone in the implementation of specific interventions.

One strength of this study is its presentation of a segmentation of targets aimed at promoting the dissemination of hepatitis virus testing workplace. To the best of our knowledge, this is the first study to apply correspondence analysis to segmentation in social marketing in public health. Various methods have been used for segmentation in various health communication campaigns (35). A Korean study that pinpointed targets for public health interventions during the COVID-19 pandemic utilized descriptive statistics to identify three groups through hierarchical cluster analysis (36). The results suggest a recommendation for segmentation focusing on the poverty of social and media resources, rather than on the demographics of the target population. In Australia, segmentation in a vaccination campaign for COVID-19 was conducted using latent class analysis based on questionnaire survey responses about perceptions and attitudes toward vaccines in the framework of the theory of planned behavior (37). The authors identified different trusted experts and information sources in each group. Correspondence analysis would be unique because it visualizes several categorical variables simultaneously, making it easier to interpret than other methods. Further evaluation of the superiority of these segmentation methods requires further study.

This study has some limitations. First, objective data were not available since the information was collected through a self-administered questionnaire. Collecting objective information, such as data on the subject’s position and number of media accesses, is important for future research. Second, we could not verify whether this strategy would result in widespread hepatitis virus testing in the workplace. We intend to conduct further research on implementing hepatitis virus testing in the workplace in these media. Herein, we aimed to verify the effectiveness of this strategy based on the number of visits to our articles, text data of comments, and reader surveys. Third, we did not conduct random sampling. We must consider that the respondents in this study have a high level of Internet literacy and characteristics that favor surveys.

## 5 Conclusion

We conducted a segmentation analysis to identify the priority group for promoting hepatitis virus testing in the workplace. Based on the findings of this study, disseminating the necessary information through appropriate media is considered an effective strategy. This study offers important recommendations for effectively delivering information that captures the attention of interested individuals in an information-saturated society.

## Data Availability

The raw data supporting the conclusions of this article will be made available by the authors, without undue reservation.
