# Supplementary material for: Marketing strategies for promoting workplace hepatitis B and C virus testing: a cross-sectional study using correspondence analysis in Japan
Source: Front Public Health. 2025 Mar 6;13:1522850. doi: 10.3389/fpubh.2025.1522850 (PMC11922932; doi:10.3389/fpubh.2025.1522850)
Supplement: Supplementary file 1 [file Table_1.docx]

# Supplementary digital contents

| Supplementary Table S1. The distribution of responses for necessary information, information media, and social networking services by positions | | | | | | | | | |  |  |  |  |
| --- | --- | --- | --- | --- | --- | --- | --- | --- | --- | --- | --- | --- | --- |
|  |  | Business Owners | | Education and Training | | General Affairs | | Labor Relations | | Other | | Recruitment | |
|  |  | N | % | N | % | N | % | N | % | N | % | N | % |
| Nessesary information | |  |  |  |  |  |  |  |  |  |  |  |  |
|  | Testing agency | 298 | 29.8 | 34 | 34.7 | 58 | 42.3 | 130 | 36.2 | 11 | 33.3 | 156 | 41.8 |
|  | Testing information | 262 | 26.2 | 34 | 34.7 | 42 | 30.7 | 102 | 28.4 | 8 | 24.2 | 152 | 40.8 |
|  | Medical facilities | 168 | 16.8 | 33 | 33.7 | 37 | 27.0 | 81 | 22.6 | 5 | 15.2 | 140 | 37.5 |
|  | Communication | 184 | 18.4 | 38 | 38.8 | 36 | 26.3 | 90 | 25.1 | 5 | 15.2 | 124 | 33.2 |
|  | Job impact | 169 | 16.9 | 27 | 27.6 | 34 | 24.8 | 78 | 21.7 | 6 | 18.2 | 109 | 29.2 |
|  | Result storage | 104 | 10.4 | 18 | 18.4 | 26 | 19.0 | 51 | 14.2 | 3 | 9.1 | 75 | 20.1 |
|  | Privacy | 101 | 10.1 | 20 | 20.4 | 20 | 14.6 | 52 | 14.5 | 5 | 15.2 | 65 | 17.4 |
|  | Prejudice management | 98 | 9.8 | 23 | 23.5 | 16 | 11.7 | 51 | 14.2 | 4 | 12.1 | 88 | 23.6 |
|  | Support plan | 96 | 9.6 | 19 | 19.4 | 20 | 14.6 | 41 | 11.4 | 2 | 6.1 | 71 | 19.0 |
|  | Treatment and work balance system | 106 | 10.6 | 11 | 11.2 | 17 | 12.4 | 49 | 13.6 | 3 | 9.1 | 54 | 14.5 |
|  | Other | 4 | 0.4 | 1 | 1.0 | 0 | 0.0 | 0 | 0.0 | 0 | 0.0 | 0 | 0.0 |
|  | Nothing in particular | 260 | 26.0 | 9 | 9.2 | 18 | 13.1 | 54 | 15.0 | 9 | 27.3 | 25 | 6.7 |
|  | Unknown | 243 | 24.3 | 15 | 15.3 | 26 | 19.0 | 88 | 24.5 | 7 | 21.2 | 52 | 13.9 |
| Media and SNS | |  |  |  |  |  |  |  |  |  |  |  |  |
|  | NIKKEI | 356 | 35.6 | 51 | 52.0 | 59 | 43.1 | 125 | 34.8 | 12 | 36.4 | 210 | 56.3 |
|  | Rodo Seisaku Jihou | 69 | 6.9 | 26 | 26.5 | 30 | 21.9 | 87 | 24.2 | 3 | 9.1 | 149 | 39.9 |
|  | Jinji-bu of Japan | 60 | 6.0 | 36 | 36.7 | 32 | 23.4 | 84 | 23.4 | 3 | 9.1 | 156 | 41.8 |
|  | HR Pro | 44 | 4.4 | 24 | 24.5 | 23 | 16.8 | 50 | 13.9 | 1 | 3.0 | 118 | 31.6 |
|  | Recruit Works | 43 | 4.3 | 18 | 18.4 | 22 | 16.1 | 47 | 13.1 | 2 | 6.1 | 118 | 31.6 |
|  | HR Mikata | 49 | 4.9 | 24 | 24.5 | 29 | 21.2 | 62 | 17.3 | 2 | 6.1 | 138 | 37.0 |
|  | BizHint | 39 | 3.9 | 18 | 18.4 | 15 | 10.9 | 32 | 8.9 | 0 | 0.0 | 112 | 30.0 |
|  | Youtube | 245 | 24.5 | 36 | 36.7 | 42 | 30.7 | 74 | 20.6 | 5 | 15.2 | 151 | 40.5 |
|  | LINE | 414 | 41.4 | 55 | 56.1 | 74 | 54.0 | 156 | 43.5 | 14 | 42.4 | 255 | 68.4 |
|  | Facebook | 264 | 26.4 | 38 | 38.8 | 34 | 24.8 | 81 | 22.6 | 5 | 15.2 | 167 | 44.8 |
|  | Instagram | 227 | 22.7 | 31 | 31.6 | 49 | 35.8 | 108 | 30.1 | 7 | 21.2 | 194 | 52.0 |
|  | X(Twitter) | 208 | 20.8 | 38 | 38.8 | 53 | 38.7 | 92 | 25.6 | 7 | 21.2 | 194 | 52.0 |

| Supplementary Table S2. The Jaccard coefficients between all information | | | | | | | | | | | |  |
| --- | --- | --- | --- | --- | --- | --- | --- | --- | --- | --- | --- | --- |
|  |  | #1 | #2 | #3 | #4 | #5 | #6 | #7 | #8 | #9 | #10 | #11 |
| #1 | Testing agency | 1.00 | 0.42 | 0.38 | 0.36 | 0.32 | 0.25 | 0.25 | 0.25 | 0.25 | 0.22 | 0.00 |
| #2 | Testing information | 0.42 | 1.00 | 0.37 | 0.39 | 0.35 | 0.26 | 0.25 | 0.25 | 0.24 | 0.24 | 0.01 |
| #3 | Medical facilities | 0.38 | 0.37 | 1.00 | 0.35 | 0.37 | 0.29 | 0.30 | 0.30 | 0.31 | 0.29 | 0.01 |
| #4 | Communication | 0.36 | 0.39 | 0.35 | 1.00 | 0.43 | 0.31 | 0.34 | 0.33 | 0.34 | 0.31 | 0.00 |
| #5 | Job impact | 0.32 | 0.35 | 0.37 | 0.43 | 1.00 | 0.30 | 0.34 | 0.35 | 0.36 | 0.33 | 0.01 |
| #6 | Result storage | 0.25 | 0.26 | 0.29 | 0.31 | 0.30 | 1.00 | 0.46 | 0.35 | 0.36 | 0.36 | 0.01 |
| #7 | Privacy | 0.25 | 0.25 | 0.30 | 0.34 | 0.34 | 0.46 | 1.00 | 0.39 | 0.38 | 0.37 | 0.01 |
| #8 | Prejudice Management | 0.25 | 0.25 | 0.30 | 0.33 | 0.35 | 0.35 | 0.39 | 1.00 | 0.41 | 0.38 | 0.01 |
| #9 | Support plan | 0.25 | 0.24 | 0.31 | 0.34 | 0.36 | 0.36 | 0.38 | 0.41 | 1.00 | 0.48 | 0.01 |
| #10 | Treatment and Work Balance System | 0.22 | 0.24 | 0.29 | 0.31 | 0.33 | 0.36 | 0.37 | 0.38 | 0.48 | 1.00 | 0.01 |
| #11 | Other | 0.00 | 0.01 | 0.01 | 0.00 | 0.01 | 0.01 | 0.01 | 0.01 | 0.01 | 0.01 | 1.00 |
